# *Strongyloides stercoralis*: Uncommon yet not to be missed cause of eosinophilia

**DOI:** 10.1002/ccr3.6653

**Published:** 2022-12-15

**Authors:** Jacob M. Boccucci, Apryl Cronley, David W. Walsh

**Affiliations:** ^1^ Medical College of Georgia Augusta University Augusta Georgia USA; ^2^ Hospital Medicine, Medical College of Georgia Augusta University Augusta Georgia USA

**Keywords:** blood, epidemiology, parasitology, pathology

## Abstract

*Strongyloides stercoralis* is a soil‐transmitted nematode that is estimated to infect millions of people per year worldwide. However, cases are less commonly seen in the United States. This report highlights the importance of when to include *Strongyloides stercoralis* in the differential diagnosis of patients presenting eosinophilia within the United States.

## Graphical abstract

*Strongyloides stercoralis* is a soil‐transmitted nematode that is not commonly diagnosed in the United States. *Strongyloides stercoralis* should be included in the differential diagnosis of eosinophilia to ensure appropriate and timely treatment.

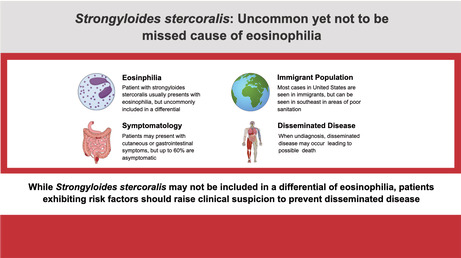

## 1 INTRODUCTION

*Strongyloides stercoralis* is a soil‐transmitted nematode that is estimated to infect between 3 million and 100 million people per year worldwide, particularly in South East Asia, African, and Western Pacific regions of the world.^1^, ^2^, ^3^ In the United States, the majority of confirmed cases are seen in immigrants.^4^ However, cases have been seen in areas of the Southeastern United States, particularly where poor sanitation provides a suitable environment for *Strongyloides stercoralis*.^4^ Patients may present with cutaneous or gastrointestinal symptoms. Additionally, patients can be asymptomatic in up to 60% of cases. In such cases, often the only clinical clue is eosinophilia. An asymptomatic carrier state increases the risk of developing *Strongyloidiasis* hyperinfection syndrome leading to systemic sepsis and end organ failure.^5^ Due to the overall low prevalence of disease in the United States and its unique geographical distribution within the United States, physicians may overlook *Stronglyoides stercoralis* as a possible diagnosis. This oversight could result in the delay of appropriate treatment and adverse patient outcomes.

## 2 CASE HISTORY/EXAMINATION

A 73‐year‐old Hispanic man with a significant medical history of alcohol dependence, thiamine deficiency, and B12 deficiency is brought to the emergency department by his ex‐wife due to abnormal behavior. She states that the patient has been talking to himself and hallucinating, making statements like “there are dead people inside me.” Per secondary report, the patient has been experiencing audiovisual hallucinations and responding to internal stimuli, often seen talking to himself and swatting in the air. In addition to the psychiatric symptoms, an ROS is significant for weight loss of 20 pounds over the past 3 months. He reports decreased appetite, fatigue, and difficulty sleeping. He denied any abdominal pain, nausea, diarrhea, melena, or hematochezia. He denied cough, dysphagia, or early satiety. He admits to cocaine and methamphetamine use for the past 3 years and drinking several beers daily. Pertinent additional medical history is significant for a cerebral vascular accident (CVA) in 2020 with residual memory and cognitive deficits exacerbated by his chronic cocaine methamphetamine use. The chart review revealed no history of psychiatric diagnosis or treatment for polysubstance abuse.

On examinations, vital signs are unremarkable. Pertinent physical exam findings included a cachectic Hispanic man with poor dentition. The cardiopulmonary exam was unremarkable. Neurological exam displayed clear and coherent speech with no gross neurological deficits. He was alert and oriented to person, place, and time. He was alert and oriented to person, place, and time. A psychiatric exam revealed appropriate mood and affect.^6^

## 3 DIFFERENTIAL DIAGNOSIS, INVESTIGATION, AND TREATMENT

**FIGURE 1:**
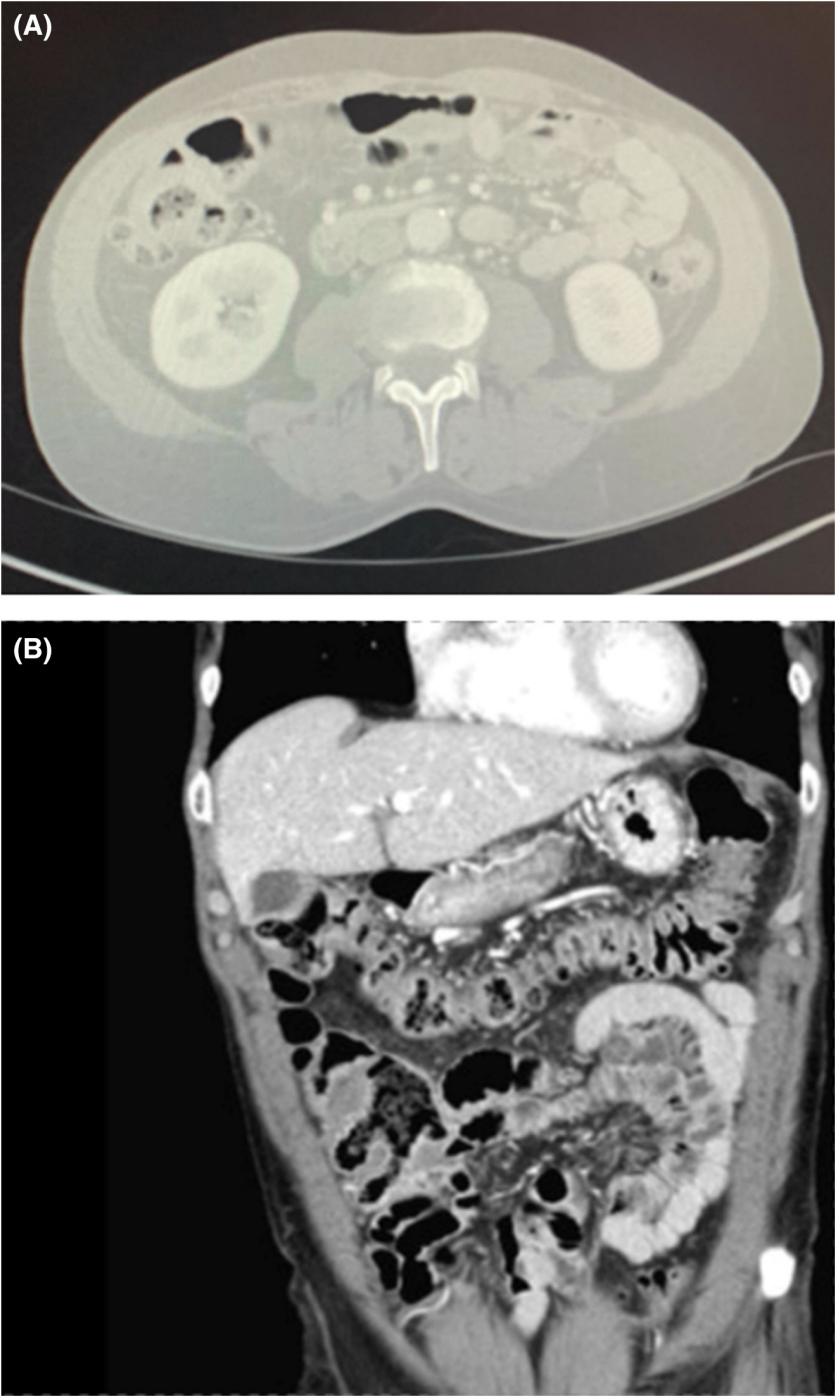
(A). Computed tomography (CT) axial view of abdomen showing nonspecific wall thickening in the cecum and proximal ascending colon. (B). Computed tomography (CT) coronal view of abdomen showing nonspecific wall thickening in the cecum and proximal ascending colon

A workup was conducted for acute polysubstance abuse with a urine drug screen revealing the patient was positive for cocaine and amphetamine use. Computed tomography (CT) without contrast was taken of the head revealing chronic microvascular changes but no acute intracranial process. Due to chronic alcohol intake, the patient was started on folate and thiamine supplementation. Initial complete blood count (CBC) revealed a hemoglobin count of 12.9 and a platelet count of 145. With recent weight loss and malnutrition, a CT scan of the chest, abdomen, and pelvis was obtained to evaluate for malignancy. Scans revealed a 3 mm right upper lung (RUL) nodule and nonspecific wall thickening in the cecum and proximal ascending colon (Figure 1). It was recommended at that time to have a follow‐up CT scan in 12 months and a referral for outpatient colonoscopy. White blood count (WBC) was elevated at 13.0 (ref 4.8–10.8) with differential revealing eosinophil percentage of 22% (ref 0–4) and absolute (ABS) eosinophil count of 2.9 (0–0.4). Chart review discovered chronic eosinophilia since 2013. Hematology was consulted for the evaluation of eosinophilia. HIV test, malignancy workup to include peripheral blood smear and FISH, and infectious workup including Strongyloides antibody were ordered, which were pending at the time of discharge.

## 4 OUTCOME AND FOLLOW‐UP

The patient was discharged on vitamin supplementation along with follow‐up with primary care, mental health, and gastroenterology. One week following discharge, Strongyloides antibody returned positive, and patient was informed of his diagnosis and prescribed Ivermectin. The patient followed up with infectious disease and finished his course of treatment. At 3‐month follow‐up, patient's absolute eosinophil count decreased from 2.9 to 1.2, and he reported a resolution of shortness of breath, diarrhea, and abdominal pain.

## 5 DISCUSSION

Eosinophilic disorders include a broad differential of etiologies with presentations ranging from asymptomatic and incidental findings upon routine evaluation to fulminant and fatal outcomes. Eosinophilia is defined as an upper limit of absolute eosinophil count (AEC) around 500/mm^3^ to 1500/mm^3^ while hypereosinophilia is defined as an AEC greater than 1500/mm^3^.^7^ Eosinophilia can further be classified as either primary or secondary. Secondary causes are more common and include etiologies such as infection, hematologic malignancies, and allergies.^3^ Due to the heterogenic and, at times indolent, nature of eosinophilic‐related diseases, certain diagnosis can often be overlooked. Such was possibly the case in this patient with *Strongyloides stercoralis* infection.

When a patient presents with eosinophilia, the first step in evaluation after excluding signs of end‐organ damage is to exclude common causes of eosinophilia. This includes workup for allergies, infections, and autoimmune disorders.^5^ After laboratory confirmation, treatment should be promptly initiated. However, if basic workup returns negative, a hematology consult should be considered to evaluate for other causes, such as primary hyperinfection syndromes.

Due to the low prevalence of *Strongyloides stercoralis* in the United States, particularly in nonimmigrant Americans, diagnosis can be easily missed. The majority of patients will be asymptomatic other than peripheral eosinophilia.^7^ If symptomatic, patients may present signs of infection that include dyspnea, abdominal pain, and diarrhea. Further, some patients, will present with disseminated Strongyloides, which includes shock, disseminated intravascular coagulation, meningitis, renal failure, and/or respiratory failure. Disseminated Strongyloides is a difficult diagnosis to establish and requires a high level of suspicion.^4^ Patients who are at high risk of fulminant disease include those who are immunocompromised, those have traveled to an endemic region, or those affected by poor sanitation.^8^

While clinicians may not suspect *Strongyloides stercoralis* in their initial differential of eosinophilia, we hope to demonstrate the importance of clinical suspicion in a patient with risk factors. This not only includes immigrants and immunocompromised patients but also can be seen in patients with chronic alcoholism and environments with poor sanitation. This was the case in our patient. Prompt treatment in patients with positive serologic evidence of Strongyloides with Ivermectin is important in preventing disseminated disease and potentially fatal outcomes.

## AUTHOR CONTRIBUTIONS

AC and JB cared for the patient, conducted research, and prepared the manuscript. DW assisted with the preparation of the manuscript, case review, and research review.

## CONFLICT OF INTEREST

The authors have no conflicts of interest to declare.

## ETHICAL APPROVAL

Written informed consent was obtained from the patient to publish this report in accordance with the journal's patient consent policy.

## CONSENT

Published with the consent of the patient.

## Data Availability

Data sharing not applicable to this article as no datasets were generated or analyzed during the current study.
